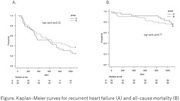# Impact of dementia on the prognosis of elderly patients with heart failure

**DOI:** 10.1002/alz70860_100660

**Published:** 2025-12-23

**Authors:** Manabu Kokubo, Akihiro Hirashiki, Takahiro Kamihara, Hidenori Arai, Atsuya Shimizu

**Affiliations:** ^1^ The National Center for Geriatrics and Gerontology, Obu, Aichi, Japan; ^2^ National Center for Geriatrics and Gerontology, Obu, Aichi, Japan

## Abstract

**Background:**

Elderly patients with heart failure frequently suffer from cognitive impairment. It is generally believed that patients with heart failure and dementia are more likely to experience events such as rehospitalization and death than those without dementia. On the other hand, drug treatment for heart failure has continued to advance for over 20 years, improving the prognosis. In this study, we aimed to determine whether such advances in treatment methods have improved the prognosis of elderly patients with heart failure and cognitive impairment.

**Method:**

This retrospective study targeted patients aged 80 to 89 years who were hospitalized for the first time for heart failure between 2018 and 2021. They were divided into dementia and non‐dementia group, and were followed up for three years to investigate the occurrence of events such as rehospitalization and death (Approval no. 1272).

**Result:**

There were 85 cases that met the criteria for this study (35 in the dementia group and 50 in the non‐dementia group). The age at first hospitalization for heart failure tended to be higher in the dementia group, but the difference was not significant (dementia group 85.0±3.1, non‐dementia group 83.8±3.5, *p* = 0.09). There were no significant differences between the two groups in terms of patient background, such as gender ratio, complications, or number of medications being taken. In addition, hypertension and atrial fibrillation were common as underlying diseases in both groups. In the non‐dementia group, 82% had heart failure with preserved ejection fraction (HFpEF), while in the dementia group, 69% had HFpEF and the proportion of heart failure with reduced systolic function (HFrEF) was increasing (*p* = 0.025). There were no significant differences between the two groups in re‐hospitalization due to recurrent heart failure and total mortality during the 3‐year follow‐up period (long‐rank *p* = 0.31, long‐rank *p* = 0.77).

**Conclusion:**

The three‐year follow‐up in this study suggested that the prognosis of elderly patients with heart failure and dementia was almost equivalent to that of elderly patients with heart failure without dementia.